# A Simulation Workshop for Pediatric Residents Using Team-Based Reflective Practice to Communicate Life-Altering News

**DOI:** 10.7759/cureus.22695

**Published:** 2022-02-28

**Authors:** Nino Rainusso, Daniel Lemke, Ernest Frugé

**Affiliations:** 1 Pediatric Oncology, Texas Children's Hospital, Baylor College of Medicine, Houston, USA; 2 Pediatric Emergency Medicine, Texas Children's Hospital, Baylor College of Medicine, Houston, USA; 3 Pediatrics, Texas Children's Hospital, Baylor College of Medicine, Houston, USA

**Keywords:** simulation, team-based, pediatrics education, life-altering news, reflective practice

## Abstract

Introduction

Guiding patients and their families through threat and tragedy is an essential skill for physicians. Educational opportunities to acquire this crucial expertise during medical training are limited. We describe a workshop design employing simulation and team-based reflection to enhance pediatric residents' confidence in delivering life-altering news.

Methods

Three hundred and seventy-six pediatric residents participated in an annual 2.75-hour workshop from 2011 to 2018. For each session, 24 to 28 residents were randomly assigned to learning teams of 6-7 trainees and two faculty. Each team had four different simulated parent encounters to convey life-altering news. Briefing and debriefing of encounters utilized team-based reflection. The impact of this educational intervention was evaluated using retrospective pre-post self-report questionnaires.

Results

Participants indicated that the learning experience was realistic, useful, and was provided in a safe learning environment. Residents reported increased confidence in their ability to communicate various types of life-altering news. A one-year follow-up survey indicated most respondents found the experience useful in actual practice subsequent to the workshop. The design also appears to be an efficient use of resources.

Conclusion

A workshop combining team-based reflection and simulation improves the confidence of pediatric residents in communicating life-altering news.

## Introduction

The definition of life-altering or bad news in the context of medical practice includes any information provided by physicians that significantly affects the behavior, emotions, or cognitive processes of patients and their families [1]. The impact varies depending on a number of factors, including immediate consequences, longer-term risks, and concurrent psychosocial issues (e.g., life-threatening conditions, serious medical error, economic hardship, family dysfunction). Practicing physicians and trainees regularly encounter these complex and difficult situations. Delivering life-altering news produces a significant emotional and psychological load on health care providers. Sources of strain include incomplete knowledge of prognosis, uncertainty about the patient/family reaction, and inadequate understanding and management of the provider's own emotions. Physicians' lack of adequate training for these types of situations contributes to this burden [2,3].

Reflection in and on practice helps providers improve their capacity to consciously assess and manage their emotions, maintain disciplined reasoning, and guide patients with compassion and flexibility through circumstances where there is no clear solution [4]. In addition, reflection can enhance the physician's pursuit of lifelong learning and appears to be suitable to and stimulated by complex clinical problems [5-8].

Guiding patients and their families through times of threatening circumstances is a crucial skill for physicians. These situations are often complex and emotionally charged for all concerned, requiring the physician to thoughtfully tune their approach to the unique patient/family responses. To address this problem, we designed a workshop for pediatric residents to enhance their confidence in the delivery of life-altering news. This workshop combined challenging simulated encounters with opportunities to reflect on past experiences in teams facilitated by seasoned clinicians. We hypothesized that participant's reflection would enhance their confidence in communicating life-altering news to patients' families. The impact of this intervention was evaluated using 7-point Likert-type questionnaires.

Preliminary data from this project was presented as a poster at the Pediatric Academy Societies Annual Meeting, Vancouver, British Columbia, Canada, May 3-6, 2016.

## Materials and methods

Between 2011 and 2018, all second-year pediatric residents participated in a "Delivering Life-Altering News" workshop at Texas Children's Hospital, a quaternary care facility in Houston, Texas. Post-call residents or those on vacation were not required to attend. The activity was part of an annual resident professional development day. Baylor College of Medicine's Institutional Review Board determined that our educational project was exempt research.

We presumed that second-year pediatric residents had, by that point in their training, witnessed or directly participated in a variety of situations where life-altering news had been communicated to patients and their families. The 2.75-hour workshop included a brief orientation as to the purpose, schedule, and logistics of the workshop (e.g., room locations) but no didactic presentations. The potential of simulated encounters to evoke memories of analogous practice experiences was seen as a crucial element of the design. The encounters included: i) new diagnosis of leukemia, ii) diagnosis of ambiguous genitalia, iii) discussion of the impending death of a patient, and iv) disclosure of an adverse event (see appendices). These cases were selected and extensively reviewed every year by the authors based on their clinical experience (Nino Rainusso and Daniel Lemke). During the orientation, the following points were emphasized: 1) the workshop was designed to help all participants reflect on their practice of guiding others through threat and tragedy, 2) there was no evaluation of the residents' performance or checklists of the target behavior, only collective reflection on practice and discussion in teams, 3) all events in the workshop were confidential (not disclosed to external parties). 

Prior to the workshop each year, actors simulating the role of parents (standardized parents or SPs) were provided with detailed case descriptions, the range of typical emotional reactions to these situations, and rehearsals with feedback from faculty. The SPs usually had substantial prior experience portraying simulated patients or parents. For this design, the actors were specifically directed to not track or evaluate the residents' specific behaviors. Rather, SPs were given latitude to respond during encounters in whatever way seemed congruent with their understanding of the character they were portraying, the situation, and the residents' specific approach. The objective was to maximize a sense of realism in the encounter. The workshop itself was staged at the Texas Children's Hospital Simulation Center.

There were two workshop sessions each year to accommodate the entire class. For each session, 24 to 28 residents were randomly assigned to four learning teams, each with 6-7 trainees and two facilitating faculty. The faculty were primarily academic general pediatricians and pediatric hematologists/oncologists with a smaller number of other pediatric sub-specialists. Each faculty member had gone through an orientation about the briefing and debriefing methodology prior to attend the workshop. A lawyer from the Baylor College of Medicine Office of Risk Management observed and participated in all "adverse event" encounter discussions. His role was to provide information about institutional risk management policies and procedures related to this scenario. He also joined a final plenary session as a resource for questions that may arise at the end of the workshop. Three to four administrative staff and faculty functioned as "managers" to help keep time and coordinate the flow of events.

Four residents in each learning team were randomly selected to conduct one of the encounters as representatives of the team. Therefore, not all residents had individual encounters with the SPs. Each encounter had three phases. In the 10-minute preparation (briefing) phase, the team reviewed the information provided for each scenario. Faculty facilitated the team's "anticipatory reflection" as to the task at hand, including consideration of potential parental concerns and reactions. In addition, alternative management strategies were discussed based on the residents’ collective reflection on past clinical experiences. The faculty did not provide specific guidance on how to approach the encounters.

The second phase was a seven-minute encounter between a resident representative and an SP. The encounter was observed via video by the other members of the resident's learning team. The observers were encouraged to reflect upon their own comparable clinical experiences, including parental responses as well as their own reasoning, emotional reactions, management strategies, and associated outcomes in those situations. The faculty avoided making any comments during the encounter. However, faculty could stop the scenario at any time if they thought it was too stressful for the resident representative. 

The third phase was a 10-minute debriefing discussion. The resident representative and SP joined the observing learning team. Both were asked to briefly describe their experience of the encounter. In contrast to traditional simulated patient encounters, the SPs in this design had been instructed to not critique the performance of the representative, but instead direct their comments to the learning team as a whole. They were asked to describe, to the best of their ability, their experience in the parent's role and how they as a parent, might respond in such situations. For example, if the SP thought a resident was evasive in discussing the specifics of prognosis, rather than addressing the representative by saying "You said …", the SP might say, "In these situations, I think a parent would be very frightened and anxious about the risk to their child's life and might want a physician to …". This was designed to consistently move the learning team discussion away from a focus on the representative's behavior to the observing members' reflection on their own practice experience. 

Likewise, the faculty were also trained to redirect the discussion from a review of the representative's behavior to the team's reflections on their own memories of previous clinical situations evoked by observing the encounter and how this reflection might inform their future practice. The instructors were welcomed to disclose information about their own comparable experiences if they judged this would facilitate the team's reflection. Examples of faculty facilitation statements are included in Table 1 (see also appendices for additional information about the method). The time for each encounter was initially based on an estimate by the authors as to how much time would be required to adequately evoke memories of comparably complicated and emotionally charged scenarios. Although the time may seem short, it has proven to be reliably and remarkably adequate across the years of the workshop. 

**Table 1:** Examples of suggested questions and comments for faculty facilitation of learning teams * Questions or comments addressed to the whole learning team SP - standardized parents

Phase	Questions
Preparation (briefing) phase*	“The purpose of observing these encounters is to help you remember similar cases you have experienced in your practice, and to reflect on what you thought, felt and did in those situations.” “Have you encountered similar situations in the past? How did you approach those discussions?” “How do you think the mother might respond in a situation like this?” “What do you think should be the focus of your discussion with the patient’s father?” “Are there any specific words that you might use to describe…?“ “Are there any words or phrases you would like to avoid?”
Debriefing phase*	“Did watching this encounter bring any similar situations from your own practice to mind?” Follow-up questions for encounters recalled by residents: “Do you recall how you felt during those encounters?” “Did your emotions effect your approach?” “How did you manage those emotions?” “Do you recall what you thought during those encounters?” “How did your thinking effect your approach?” Faculty self-disclosure of reflections on similar cases: “As I watched, I thought about how it is still difficult for me to… (e.g. say those words, control my irritation, tolerate silence, etc.) “After observing this encounter and thinking about your own practice, what might you do differently next time you are in a similar situation?”
Phrases to avoid	“I thought that you had a great/awful interaction with the SP” “You forgot to mention that ….” Or “You did not include…” “Everybody knows that difficult conversations about life-altering news should be guided by the SPIKES mnemonic.” "You must include… in your conversation with parents.”

After completing the three phases described above, the learning teams, including facilitating faculty, would rotate to a different room and begin the cycle of preparation, encounter, and debriefing again with the next case. When the learning teams had completed the four cases, all participants, including the actors, gathered for a summary review of the entire process. Particular attention was given to the discussion of lingering questions or key insights that emerged over the course of the workshop. Finally, residents completed a simple retrospective pre-post evaluation of the workshop. Specifically, residents were asked to rate changes as to their confidence in communicating various types of life-altering news as a result of participating in the workshop (seven-point Likert type scale ranging from strongly disagree to strongly agree). Data was analyzed using a paired t-test.

We attempted to collect follow-up information about one year after the residents' participation in the workshop to determine if the workshop had any long-lasting impact on their confidence in delivering life-altering news. This was typically done during a routine lecture where a substantial number of the residents were likely to be present. The survey simply asked the residents if they had attended the workshop and, if so, to estimate the number of times they had directly communicated bad or life-altering news to families in the months since the workshop. They were also asked to rate the utility of the workshop in those scenarios. All questions but one (i.e. the sudden and unexpected death of a patient) were directed to evaluate the residents' confidence in communicating life-altering news in clinical situations similar to the cases encountered during the workshop.

## Results

Over a period of eight years, 376 second-year pediatric residents participated in the workshop (85% of a possible 442 participants). Residents indicated that the workshop design provided a learning experience that was realistic, useful, and conducted in a not unduly stressful environment (Table 2, general appraisal of workshop experience). Residents reported a significant improvement in their confidence in communicating life-altering news for all assessed tasks (Table 2, self-reported confidence in communicating life-altering news). The evaluation did not differentiate between residents who were team representatives for encounters and those who were observers. 

**Table 2:** Workshop participants' self-reported outcomes # Retrospective questionnaire, seven-point Likert type scale (7=strongly agree) † Retrospective pre-post questionnaire, seven-point Likert type scale (7=strongly agree) * p<0.001

General appraisal of workshop experience (n=376)	Mean^#^ (SD)	
During these simulation exercises I experienced a sense of realism	6.1 (1)	
The debriefing sessions were useful without being unduly stressful	6.2 (0.9)	
The course was appropriate for my level of learning	6.4 (0.8)	
The simulation center equipment, staff, and space provided an effective learning environment	6.6 (0.7)	
I feel that my instructors provided a safe and non-threatening environment for learning	6.7 (0.6)	
I feel that my instructors provided adequate opportunity for questions and discussion	6.7 (0.6)	
Self-reported confidence in communicating life-altering news (n=376)	Pre-workshop mean^†^ (SD)	Post-workshop mean^†^ (SD)
I am familiar with the basic principles of how to break bad news to patients, parents and relatives of patients	5.2 (0.9)	6 (0.6)*
I know how to break the news to a parent (who I do not know well) that their child had suddenly and unexpectedly died	3.8 (1.2)	4.6 (1.2)*
I know how to break the news to a parent (who I know well) that their child will die after years of struggling with a chronic illness	4.6 (1.1)	5.4 (1)*
I know how to break the news to a parent that there has been a medical error that has caused serious harm to their child	4.3 (1.1)	5.6 (2.2)*
I know how to respond to a parent who reacts in a very hostile fashion after receiving bad news concerning their child	4.5 (1.1)	5.5 (1)*
I know how to respond to a parent who is overwhelmed by emotion upon hearing bad news concerning their child	4.9 (0.9)	5.8 (0.7)*
I know how my own emotional response can affect the way I deliver bad news and I know how to work with these emotions	5.1 (1)	5.7 (0.9)*

A sample of 136 residents (36% of total workshop participants) completed the one-year follow-up survey. In the intervening period, the vast majority of surveyed participants had to communicate life-altering news to parents that reacted in a hostile fashion or parents who felt overwhelmed (Table 3). The residents felt that the workshop prepared them well to deal with those situations. In general, the majority of participants thought the workshop had a positive impact on their confidence in delivering life-altering news in comparable settings. Interestingly, this effect also was perceived, although to a lesser degree, in a scenario that was not specifically included in the workshop (i.e., the sudden and unexpected death of a patient).

**Table 3:** One-year follow-up survey of workshop utility in practice (n=136) *Agree to strongly agree on a seven-point Likert scale (strongly disagree=1, strongly agree=7) # Seven-point Likert scale (strongly disagree=1, strongly agree=7)

	Number of residents who encountered a specific scenario (first column in bold) during the year following the workshop	Number of those residents rating the workshop as useful in those specific scenarios*	Mean rating of workshop utility by those residents in these specific scenarios^#^
The workshop improved my ability to break the news to a parent (who I did not know well) that their child had suddenly and unexpectedly died	20 (15%)	14 (70%)	4.9 (SD±1.2)
The workshop improved my ability to break the news to a parent (who I knew well) that their child had died (or would soon die) after years of struggling with a chronic illness	26 (19%)	21 (81%)	4.9 (SD±1.2)
The workshop improved my ability to break the news to a parent that there had been an adverse event or medical error that had caused serious harm to their child	44 (32%)	40 (91%)	5.8 (SD±1)
The workshop improved my ability to respond to a parent who reacts in a very hostile fashion after receiving bad news concerning their child	100 (74%)	84 (84%)	5.3 (SD±1.1)
The workshop improved my ability to respond to a parent who is overwhelmed by emotion upon hearing bad news concerning their child	121 (89%)	106 (88%)	5.5 (SD±1)

Finally, we also found that the workshop design was an efficient use of resources. We estimate that a design that would require all learners to personally engage in all encounters and to receive individual feedback would require 4-5 times more resources (e.g., faculty, facility, and actor time; see table 4 in appendices). 

## Discussion

Receiving life-altering news is always difficult for the patient and family. It is also a significant challenge for the medical provider. A survey of Canadian program directors and residents found they agreed that interactive workshops are the most suitable educational approach for learning how to break life-altering news in pediatrics [9]. Although there are a variety of established methods for this purpose, we think our procedures for facilitating team-based reflection in combination with the use of simulated parents have unique features. Our findings suggest that residents' confidence to effectively perform this crucial task is enhanced by this approach and could be used in real-life scenarios, and these outcomes are achieved with efficient use of the resources required to produce high-quality simulation events. 

Simulation has proved to be a very effective teaching modality where there are technically complex situations that require specific, evidence-based actions [10,11]. Often, a pre-set checklist of skills and behaviors that are considered desirable form the basis of the evaluation [12,13]. Our workshop design differed from other approaches in several key ways. Primarily, the strategies to communicate life-altering news developed by the learning teams were not prompted by explicit reference to any of the well-established guidelines for the delivery of bad news, such as SPIKES or ABCDE [14,15]. In contrast, strategies arose from the learning teams' facilitated discussions during the preparation phase. Secondly, the facilitation of debriefing discussions was intended to promote learning through reflection on and critical review of collective experiences rather than the evaluation of an individual's performance according to a priori standards. Moreover, we believe that the long-lasting effect in the residents' confidence observed one year after their participation in the workshop may be related to our methodology using reflection in the preparation and debriefing sessions. Interestingly, the late stage of professional development in physicians ("master") is characterized by the incorporation of reflection in their practice as proposed by Dreyfus and Dreyfus and described by Carraccio et al. [16,17].

Reflection is a "conscious, active and deliberate" thinking process elicited by complex situations when the "best" course of action is uncertain [4,5]. Reflection in and on practice is thus a core skill that promotes a physician's ability to tune their approach to the unique, complicated, and often emotionally intense patient/family encounters associated with life-altering circumstances. Our workshop design contained many of the triggers for reflection identified by Plant et al. [18]. The cases in our workshop exposed learners to uncertainty regarding medical knowledge or patient care, unfortunate clinical outcomes, and challenging interactions.

The results are also congruent with prior studies that found observers of simulated encounters also can learn, particularly if their roles as observers are well defined [19]. The residents indicated that they experienced the scenarios as realistic and that the learning environment was safe and effective. The overall low costs of producing such team-based simulation designs might increase access to this type of educational opportunity. Finally, we believe the team-based approach encourages the learners' capacity for "team reflexivity", a capability that can enhance the collective competence of teams for the task of guiding patients and families through life-altering circumstances [20,21].

Our study did have a number of limitations. All participants in this project were from a single institution, and the outcome measures relied upon simple self-report. Moreover, only 36% of workshop participants completed the one-year follow-up survey, which may mean that residents who thought the workshop was useful were more likely to respond. The impact on clinical practice and patient care was also not evaluated. The next steps will include the identification of common themes explored by the trainees during the preparation and debriefing phases and the comparison of this method of debriefing with other traditional methods.

## Conclusions

We developed an innovative, time-efficient workshop to enhance the communication skills of pediatric residents for the delivery of life-altering news. The design involved randomly assigning residents to learning teams that engaged in and reflected on four different simulated parent encounters. Our workshop departed from traditional approaches in that there were no pre-determined target behaviors, no didactics, and no evaluation of individual residents' performance, only team-based reflection on practice. We found that our intervention significantly improved pediatric residents' confidence in communicating life-altering news to patients' parents. A subsequent survey also indicated that residents found the workshop experience useful in practice during the year following the workshop. Our future research will systematically compare the learning outcomes associated with our established procedures for group debriefing (team-based reflective practice) with an alternative approach, where expert faculty share their judgments of the simulated encounters to start the group conversation. 

## Figures and Tables

**Figure 1:**
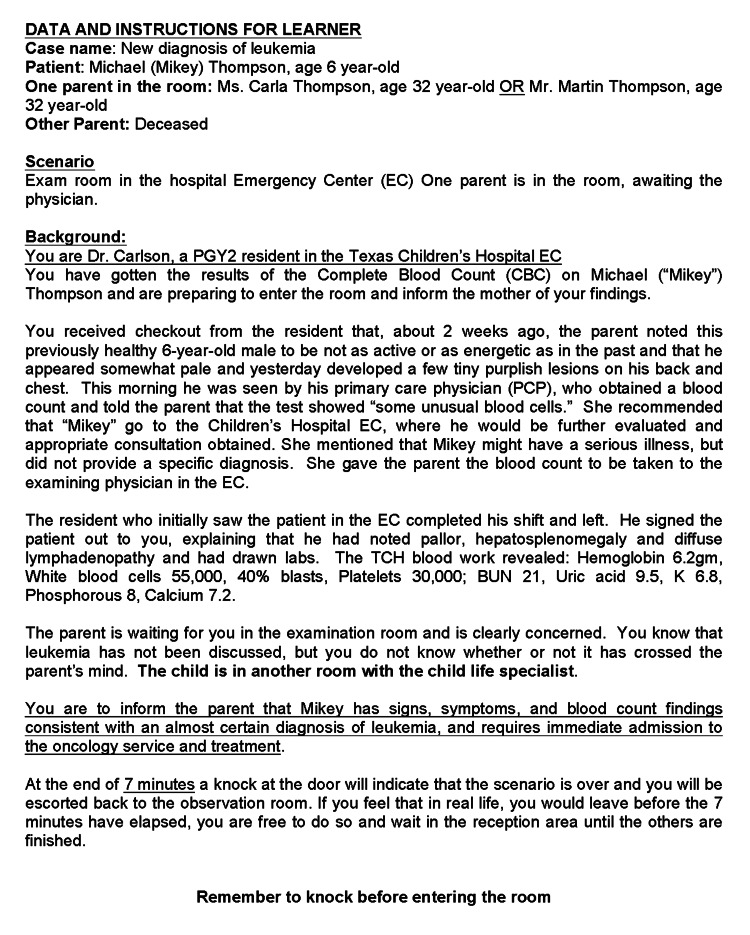
Description to learner of clinical scenario 1 Six-year-old with a new diagnosis of leukemia who has been evaluated in the emergency center.

**Figure 2:**
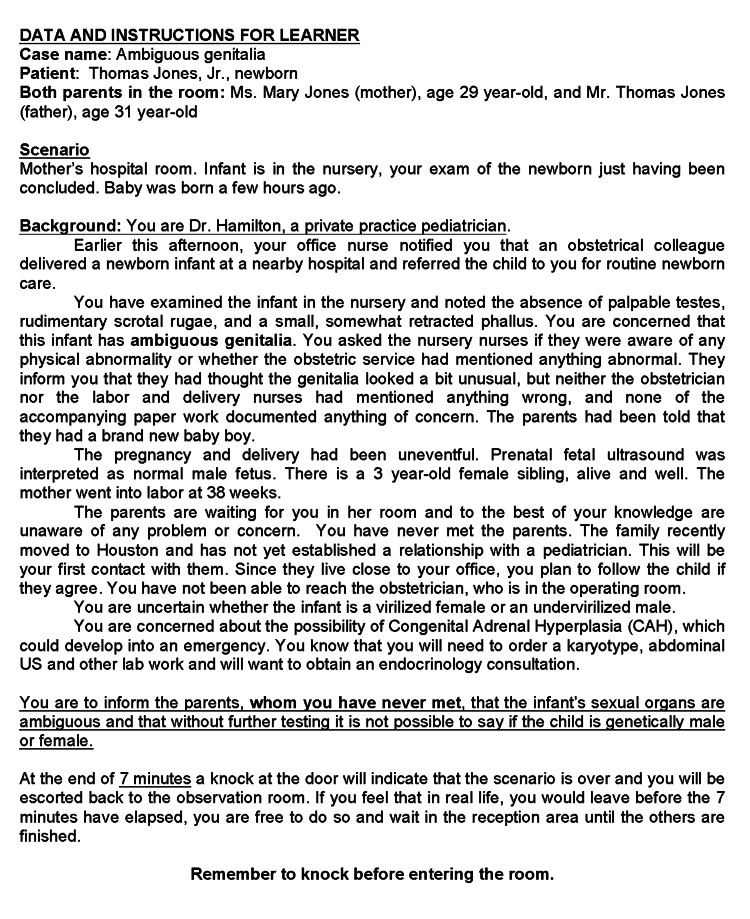
Description to learner of clinical scenario 2 Newborn baby with ambiguous genitalia.

**Figure 3:**
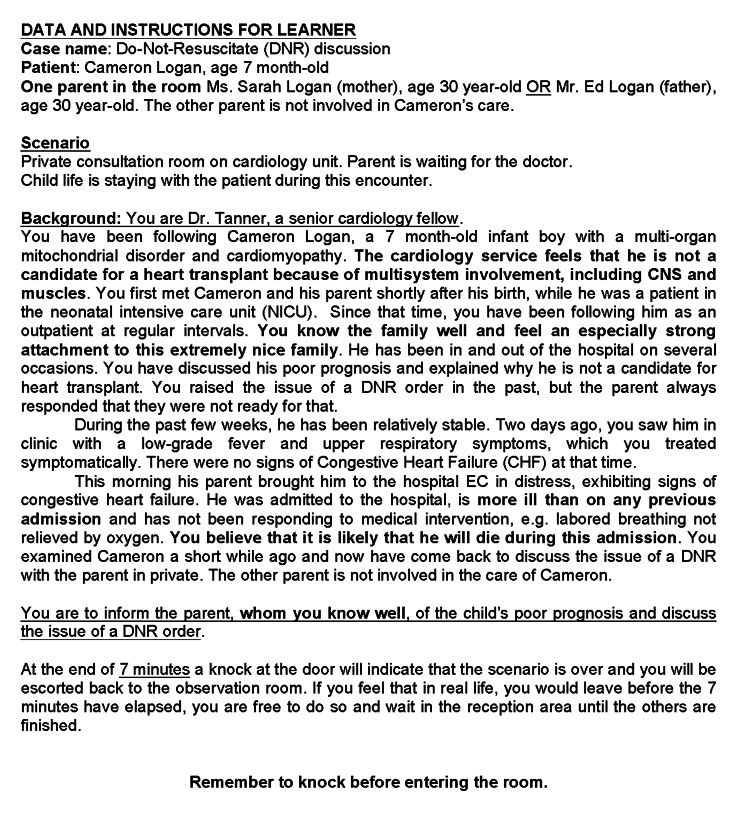
Description to learner of clinical scenario 3 Seven-month-old with multi-organ mithocondrial disorder who is admitted to the intensive care unit and probably would die during this admission.

**Figure 4:**
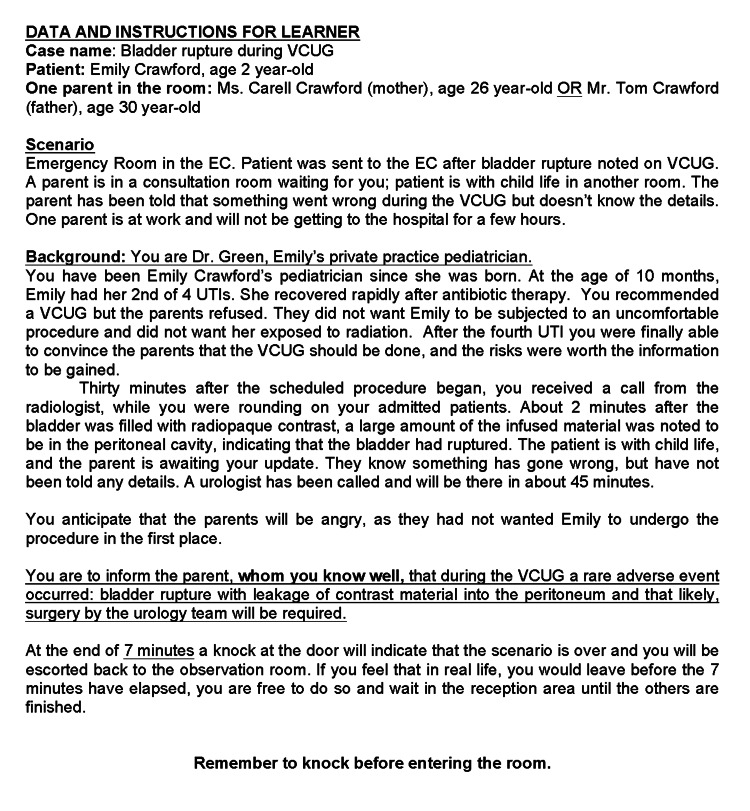
Description to learner of clinical scenario 4 Two-year-old with bladder rupture after having a voiding cystourethrogram (VCUG) study.

**Figure 5:**
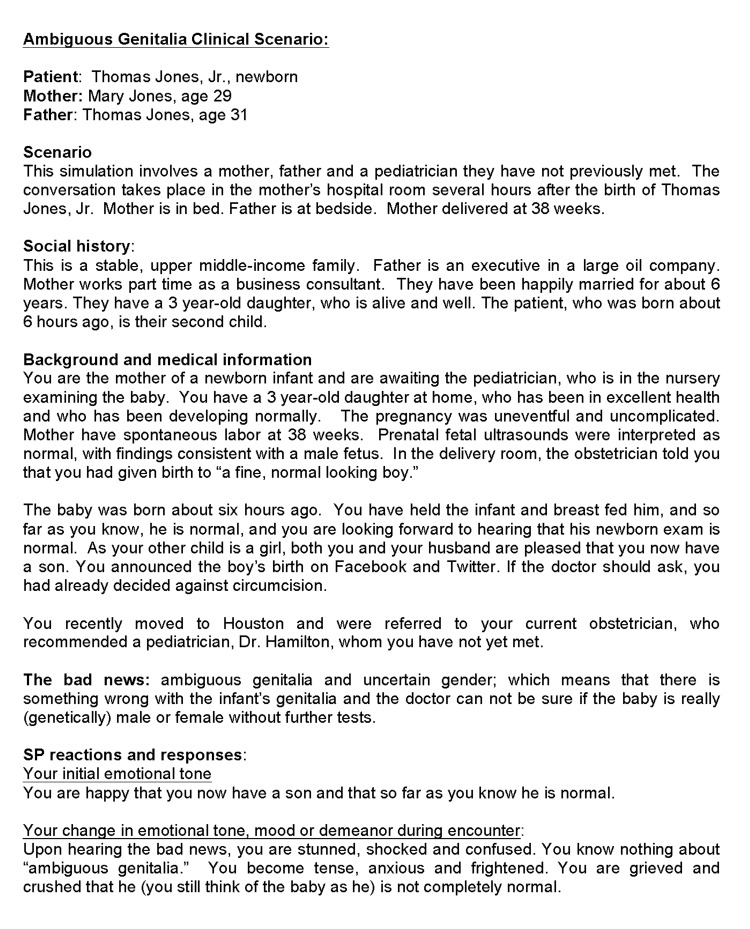
Information provided to actors portraying the role of standardized parents (SP) for the case of ambiguous genitalia (page 1 of 2)

**Figure 6:**
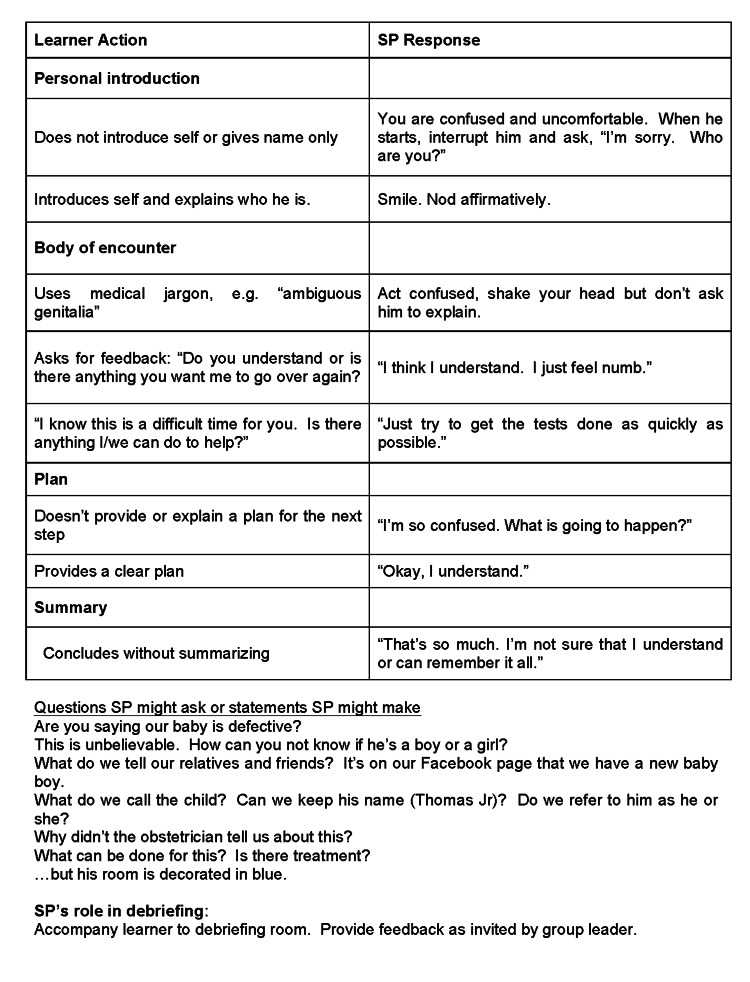
Information provided to actors portraying the role of standardized parents (SP) for the case of ambiguous genitalia (page 2 of 2)

**Figure 7:**
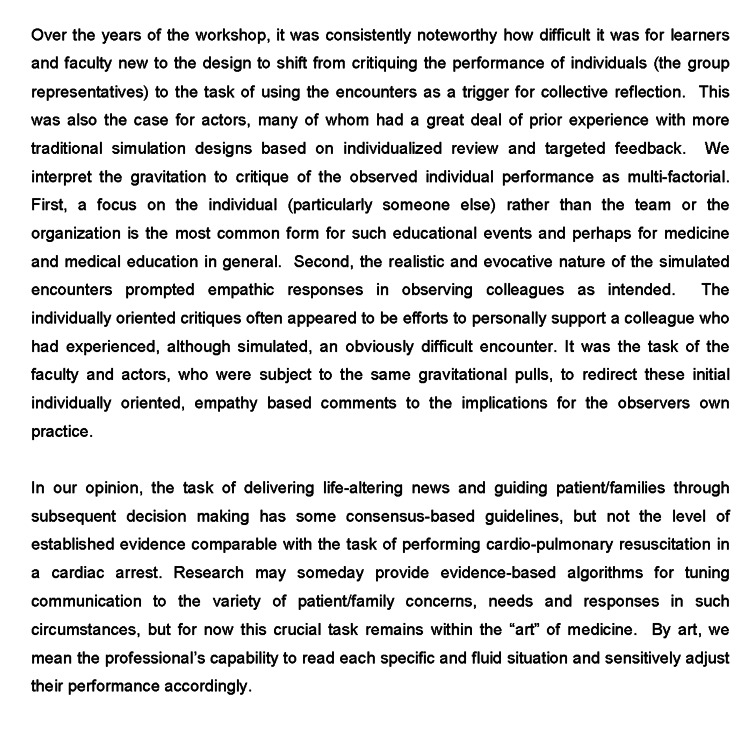
Additional information about our workshop methodology

## Appendices

**Table 4:** Time estimates of resources utilization for number of workshop participants (learners) between individual and team-based approaches

Method	Time (hours)	Learners	Learning hours	Standardized parents (hours)	Faculty (hours)	Simulation center (room hours)
Individual	11	24	264	55	88	44
Team-based	2.5	24	60	12.5	20	10
